# Emerging Perspectives in the Diagnosis and Management of Metabolic Dysfunction-Associated Steatotic Liver Disease (MASLD): A Narrative Review

**DOI:** 10.7759/cureus.95288

**Published:** 2025-10-24

**Authors:** Albine Djeagou, Kavyasri Gunukula, Anas Sermani, Sai Kiran Vaspari

**Affiliations:** 1 Health Sciences, University of Buea, Buea, CMR; 2 Medicine and Surgery, Government Medical College, Siddipet, IND; 3 Internal Medicine, Ankara Yildirim Beyazit University, Ankara, TUR

**Keywords:** diagnostic innovations, emerging therapies, metabolic dysfunction-associated steatohepatitis (mash), metabolic dysfunction-associated steatotic liver disease (masld), prognosis

## Abstract

Metabolic dysfunction-associated steatotic liver disease (MASLD, previously known as non-alcoholic fatty liver disease (NAFLD)) has emerged as the most prevalent chronic liver condition, affecting approximately 25% of the global population. The disease encompasses a spectrum ranging from simple steatosis to metabolic dysfunction-associated steatohepatitis (MASH, previously known as non-alcoholic fatty liver disease (NASH)), fibrosis, cirrhosis, and hepatocellular carcinoma (HCC). Despite its growing burden, MASLD remains under-recognized in public health discussions. This review explores the epidemiology, pathophysiology, diagnostic advancements, and management strategies for MASLD. Recent developments in noninvasive diagnostic modalities, including imaging techniques like magnetic resonance imaging proton density fat fraction (MRI-PDFF) and elastography, as well as emerging biomarkers such as CK-18 and thrombospondin-2, offer promising alternatives to liver biopsy. In terms of management, lifestyle interventions remain the cornerstone of therapy, while pharmacological approaches such as GLP-1 receptor agonists, SGLT-2 inhibitors, and FXR agonists are being actively explored. Additionally, bariatric surgery has demonstrated efficacy in select populations. Given the complex interplay between metabolic, genetic, and environmental factors in MASLD pathogenesis, a multidisciplinary approach is essential. Recent advancements in noninvasive diagnostic techniques (such as FibroScan and MRI-PDFF) for MASLD have significantly improved the assessment of liver steatosis and fibrosis. These tools, among others, have transformed the diagnostic landscape of MASLD, reducing reliance on liver biopsies. Despite these advancements, there remains a need for comprehensive reviews that integrate these diagnostic modalities with emerging therapeutic strategies, such as the increasing use of GLP-1 receptor agonists and SGLT-2 inhibitors. This review aims to bridge this gap by synthesizing current knowledge on noninvasive diagnostics and emerging treatment strategies, identifying ongoing challenges in the field, and highlighting opportunities for personalized care. This provides clinicians with an up-to-date and practical resource for managing MASLD.

## Introduction and background

Metabolic dysfunction-associated steatotic liver disease (MASLD) is a spectrum of disease defined by the shared characteristic of macrovesicular steatosis in ≥5% hepatocytes, in the absence of a secondary etiology such as alcohol or drugs. MASLD is driven by a complex “multi-hit” pathology, with the presence of inflammation being heavily correlated with disease progression to cirrhosis and hepatocellular carcinoma (HCC) [1]. The spectrum of disease ranges from the simplest form, metabolic dysfunction-associated steatotic liver (MASL), to metabolic dysfunction-associated steatohepatitis (MASH), fibrosis, and cirrhosis, with an estimated global prevalence of 25% [2,3]. MASLD is one of the leading causes of chronic liver disease worldwide, posing a significant public health challenge due to its potential progression and risk of advanced liver complications [4].

In 2023, a consensus statement by the American Association for the Study of Liver Diseases, the European Association for the Study of the Liver, and other international liver societies introduced a revised nomenclature to replace “non-alcoholic fatty liver disease” (NAFLD) with “metabolic dysfunction-associated steatotic liver disease” (MASLD) and “non-alcoholic steatohepatitis” (NASH) with “metabolic dysfunction-associated steatohepatitis” (MASH). This shift aims to better reflect the central role of metabolic dysfunction in disease pathogenesis, to reduce the stigma and confusion associated with defining the disease by alcohol exclusion, and to improve communication with patients by using terminology that is more intuitive and pathophysiologically accurate.

Despite MASLD’s increasing global prevalence, its complex pathology is still not fully understood. Additionally, the disease and its complications are widely overlooked in the public health discourse on the national obesity epidemic [4]. However, MASLD is one of the fastest-growing areas of liver research, resulting in constant advancements regarding diagnostic procedures and therapeutic options available for patients. Current projections suggest MASLD cases in the United States will increase from 83.1 million in 2015 to 100.9 million by 2030, highlighting the urgent need for effective diagnosis and treatment [3]. This review aims to elaborate on the current understanding of the disease, advancements in diagnostic techniques, and emerging therapeutic options that may significantly impact the disease's overall prognosis.

This review begins with a discussion on the epidemiology and pathophysiology of MASLD, followed by an overview of recent advancements in diagnostic procedures and management. It concludes with an analysis of prognosis and future directions.

## Review

Epidemiology

Prevalence estimates for MASLD, a significant cause of chronic liver disease globally, vary from 10% to 35%, depending on the research population and diagnostic techniques [2,5-7]. Younossi et al. analyzed 86 studies involving 8,515,431 individuals from 22 countries; the overall prevalence of MASLD was estimated to be 25.24% (95% CI: 22.10-28.65), with the lowest rates observed in Africa and the highest in the Middle East and South America [2]. Additionally, Riazi et al. estimated a prevalence of 32.4% (95% CI: 29.9-34.9) in a different analysis of 72 studies with a sample of 1,030,160 people from 17 countries. This prevalence has considerably grown over time, rising from 25.5% before 2005 to 37.8% in 2016 or later (p=0.013). Men are more likely than women to have the condition (39.7% vs. 25.6%), with 70.8 cases per 1000 person-years in men and 29.6 cases per 1000 person-years in women [6]. Younossi et al. demonstrated a rise from 25.26% (21.59-29.33) in 1990-2006 to 38.00% (33.71-42.49) in 2016-2019. Ultrasonography (US)-defined MASLD prevalence also increased by 38.7% during the same period. Latin America has the highest prevalence (44.37%), followed by the Middle East and North Africa (36.53%), South Asia (33.83%), Southeast Asia (33.07%), North America (31.20%), East Asia (29.71%), Asia Pacific (28.02%), and Western Europe (25.10%) [7].

MASLD is closely associated with metabolic comorbidities, including obesity (51.34%), type 2 diabetes mellitus (T2DM; 22.51%), hyperlipidemia (69.16%), hypertension (39.34%), and metabolic syndrome (42.54%). HCC incidence in MASLD patients is 0.44 per 1,000 person-years (range: 0.29-0.66), while liver-specific and overall mortality rates for MASLD and MASH range from 0.77 to 25.56 per 1,000 person-years [2].

Pathophysiological mechanisms

MASLD is a metabolic disorder characterized by the presence of 5% or more hepatic steatosis, as determined by liver imaging or biopsy, in the absence of secondary causes of hepatic fat accumulation [8]. It constitutes a spectrum ranging from simple steatosis, or MASL, to MASH, which is histologically defined by hepatic steatosis, inflammation, and hepatocellular ballooning with or without fibrosis [8]. A complex interaction of hormonal, dietary, and genetic variables contributes to the pathophysiology of MASLD, with IR being a key contributor [5,8,9]. Pouwels et al. suggested that there are two different types of MASLD: one closely related to metabolic syndrome, where IR is the leading cause, and another linked to external and infectious causes. Several conditions, including hepatitis C, HIV, glucocorticoids, tamoxifen, tetracycline, amiodarone, methotrexate, valproic acid, and complete parenteral feeding, can cause hepatic steatosis. Intestinal bypass surgery, genetic or acquired metabolic diseases such as lipodystrophy or cachexia, and exposure to particular toxins may also be involved [9].

Pouwels et al. also suggested that MASH is believed to develop in two phases: first, hepatic fat deposition promotes IR, followed by cellular and molecular changes that promote oxidative stress and fatty acid oxidation. Several factors contribute to these changes, including nerve injury, hyperinsulinemia, hepatic iron and lipid peroxidation, extracellular matrix remodeling, energy homeostasis dysregulation, and immune system abnormalities [9].

Role of Insulin Resistance

IR is an essential component in the pathophysiology of MASLD, causing hepatic steatosis, inflammation, and fibrosis. MASLD patients frequently show metabolic syndrome symptoms, with IR contributing to hepatic fat buildup [10]. Increased intrahepatic diacylglycerol stimulates PKC-ε and JNK1, which impede insulin signaling via insulin receptor substrates (IRS-1, IRS-2). Oxidative stress and pro-inflammatory cytokines, such as TNF-α, activate nuclear factor IKK-β, which increases hepatic IR. Given the close connection between MASLD and T2DM, IR is an important therapeutic target [10].

Hyperinsulinemia and IR contribute to hepatic fat storage by increasing de novo lipogenesis (DNL) and compromising lipid metabolism [8,11]. Normally, insulin inhibits gluconeogenesis and increases glucose absorption, but in MASLD, IR causes enhanced adipocyte lipolysis, higher free fatty acids (FFAs), and excessive hepatic triglyceride (TG) synthesis [8]. Dysregulated insulin signaling also activates sterol regulatory element-binding protein 1c (SREBP-1c) and carbohydrate response element-binding protein (ChREBP), which promote DNL and increase hepatic palmitate production. This, in turn, increases lipotoxicity, contributing to oxidative stress, inflammation, and fibrosis [5].

Role of Free Fatty Acids

Increased hepatic DNL and raised FFAs from circulation in MASLD improve fatty acid inflow into the liver [5]. While excess FFAs can be oxidized by mitochondria, peroxisomes, mitochondria, peroxisomes, and microsomes can oxidize excess FFAs. TGs are another form of FFA that can be re-esterified for storage or secretion as very low-density lipoproteins (VLDL). Hepatic steatosis occurs when VLDL secretion and oxidation are insufficient to make up for it [5,11]. FFAs that have not been esterified cause lipotoxicity, which in turn causes inflammation and cell damage [11]. It is interesting to note that suppressing TG formation worsens liver injury while lowering steatosis, and increasing TG synthesis may offer protection against FFA-induced toxicity [11].

Role of Lipotoxicity

A key contributor to the progression of MASLD is lipotoxicity, which leads to oxidative stress, inflammation, fibrosis, and IR [8]. Lipid droplet accumulation in hepatocytes is an indicator of MASLD; however, when neutral lipids cannot be stored safely, bioactive lipids build up, interfering with insulin signaling and causing cellular damage [11].

Lipotoxicity is influenced by genetic variables, specifically the PNPLA3 (rs738409, I148M) variation, which modifies hepatocyte lipid remodeling. This mutation causes excessive lipid buildup and increases liver damage by reducing PNPLA3 breakdown and impairing fatty acid metabolism [10]. Based on lipidomic investigations, MASLD is linked to a higher accumulation of free cholesterol without a corresponding increase in cholesterol esters [11]. Excess free cholesterol exacerbates hepatic damage by impairing membrane integrity, causing mitochondrial dysfunction, and increasing oxidative stress [10]. The development of the disease is accelerated by this toxic lipid milieu, which causes fibrosis and inflammation as excess free cholesterol forms cholesterol crystals in lipid droplets [10].

Role of Nutrition

Although there is little concrete evidence in people, diets high in sugars, low in dietary fiber, and high in saturated fat have all been linked to an increased risk of MASLD [8]. In addition to excessive calorie intake and subsequent weight gain, fructose, typically found in processed foods and sweetened beverages, plays a significant role in the onset and progression of MASLD [8,10]. By increasing the number of substrates available for fatty acid synthesis through the actions of aldolase B and ketohexokinase, as well as by activating transcription factors like SREBP-1c and others, fructose promotes lipogenesis [10]. It is also suggested through preclinical studies that diets high in sucrose and fructose are steatogenic because they cause an imbalance in normal intestinal microbiota and disrupt key lipid metabolic pathways and hormones [8,12].

Role of Microbiota

Dysbiosis is a key factor in MASLD, marked by reduced *Ruminococcaceae*, *Bifidobacteriaceae*, and *Faecalibacterium prausnitzii* and increased *Proteobacteria* and *Robinsoniella* [5,8,10,12]. Gut microbes influence liver inflammation, IR, and obesity, with imbalances triggering systemic inflammation via endotoxemia [10,12]. Altered liver microbiota in MASLD suggests a role in hepatic immunity. Microbiome modulation may offer therapeutic benefits [10,12]. Both gut microbiota and their metabolites influence liver inflammation and metabolism in MASLD [10]. Patients show elevated levels of short-chain fatty acids, such as butyrate, propionate, and acetate, along with ethanol produced by gut microbes, which may worsen liver damage. Obese mice show higher ethanol levels, reduced by antibiotics [10,12].

Increased gut permeability allows bacterial translocation to the liver, promoting inflammation, steatosis, and fibrosis [8,10]. MASLD patients have elevated endotoxemia and bacterial DNA, indicating systemic microbial spread [10]. LPS activates TLR4 in Kupffer cells, leading to cytokine release, fibrosis, and hepatocyte dysfunction [10,13]. Hepatic stellate cells (HSCs) drive further fibrosis, with TLR4 and MerTK mediating Kupffer cell-HSC interactions that accelerate MASLD progression [10,14-16].

Genetic Predisposition

Genetic predisposition plays a key role in MASLD development. The PNPLA3 I148M modifications (rs738409 C>G) (Table 1) exhibit a substantial association with hepatic fat storage and disease severity [5,8]. It inhibits enzymatic activity, promoting macrovesicular steatosis and raising the incidence of HCC in people with metabolic risk factors such as obesity [5,8]. Other important genetic variations include TM6SF2 (E167K), which reduces VLDL secretion and contributes to hepatic lipid storage, and TERT, which is associated with fibrosis advancement [5,8].

**Table 1:** Functional roles of gene polymorphisms in MASLD pathogenesis MASLD: metabolic dysfunction-associated steatotic liver disease, VLDL: very low-density lipoprotein, DNA: deoxyribonucleic acid, HCC: hepatocellular carcinoma

Gene polymorphism	Function	Role in MASLD
PNPLA3 (I148M, rs738409 C>G)	Triacylglycerol lipase and acylglycerol transacylase activity	Reduces enzymatic activity, leading to macrovesicular steatosis, increased hepatic fat content, and increased risk of fibrosis and HCC [5,8]
TM6SF2 (E167K)	Regulates lipid metabolism and VLDL secretion	Reduces VLDL export, promoting hepatic steatosis and fibrosis [5,8]
TERT	Maintains telomere length and regulates cellular aging	Associated with increased susceptibility to MASLD and fibrosis [5]
NCAN	Involved in lipid metabolism	Associated with hepatic steatosis and fibrosis [8]
GCKR	Regulates glucokinase activity and glucose metabolism	Variants linked to increased hepatic fat accumulation [8]
LYPLAL1	Lysophospholipase activity	Associated with hepatic fat accumulation and steatosis [8]
PPP1R3B	Regulates glycogen metabolism	Genetic variants linked to variations in hepatic fat content [8]
miR-122	Regulates fatty acid biosynthesis, lipid metabolism, and inflammation	Dysregulation contributes to MASLD progression; potential biomarker for distinguishing MASLD stages [5]
HDAC3	Epigenetic regulator of hepatic lipogenesis	Depletion leads to increased lipid synthesis and storage [5]
DNMT	Regulates DNA methylation and gene expression	Increased levels associated with fibrosis and advanced MASLD [5]

Several more loci implicated in MASLD have been found by genome-wide association studies, including NCAN, GCKR, LYPLAL1, and PPP1R3B (Table 1). All of these genetic variables modify hepatic fat storage and metabolism, explaining about 28% of the variability in hepatic steatosis [5,8]. There are ethnic disparities, with Hispanics having a higher prevalence of the PNPLA3 I148M variation and African-Americans having a higher frequency of protective alleles [8].

MicroRNAs have a substantial role in MASLD progression. The most abundant hepatic miRNA, miR-122, regulates lipid metabolism, and its dysregulation has been associated with MASH progression [5]. The expression of other microRNAs is altered, influencing inflammation, apoptosis, and cell proliferation, all of which affect illness severity. Epigenetic changes also influence MASLD progression. Reduced HDAC3 activity enhances hepatic lipid production, but aberrant DNA methylation, particularly DNA methyltransferase, is associated with illness severity [5]. Studies indicate that unique methylation patterns distinguish moderate from advanced MASLD [5,17].

Advances in diagnosis

Clinical and Laboratory Assessment

Fatty liver index (FLI): FLI is a clinically used noninvasive test for estimating hepatic steatosis. It is based on BMI, serum TGs, waist circumference, and gamma-glutamyltransferase (GGT) levels. An FLI score <30 excludes fatty liver, scores between 30 and 60 indicate uncertainty, and a score ≥60 strongly predicts the development of hepatic steatosis [18]. FLI has an AUC-ROC of 0.84 for identifying MASLD. However, its disadvantage is that it poorly distinguishes moderate-to-severe steatosis from mild steatosis [19].

Fibrosis-4 (FIB-4) index: FIB-4 is based on age, AST, ALT, and platelet count. A score ≥ 2.67 suggests advanced fibrosis, while a score ≤ 1.30 indicates low risk [20].

AST-to-platelet ratio index (APRI): APRI is less accurate than NFS or FIB-4 but can still help in fibrosis risk stratification [20]. APRI demonstrates the highest reliability for advanced fibrosis detection (AUC-ROC of 0.759), offering a strong negative predictive value of 94%. However, its limited sensitivity (55%) constrains its utility as a standalone prognostic tool [21].

Noninvasive liver fat score (NLFS): NLFS was developed using proton magnetic resonance spectroscopy (MRS) to predict MASLD and measure liver fat content. It includes AST, the AST/ALT ratio, and fasting serum insulin levels, with a threshold of liver fat >5.56%. The NLFS shows a sensitivity of 86% and specificity of 71% for detecting hepatic steatosis. It provides a cost-effective way to identify MASLD using readily available clinical and laboratory data, helping to predict patients at increased risk of the disease [18,19].

Lipid accumulation product (LAP): LAP was developed to identify fatty liver disease and assess cardiovascular risk by categorizing patients into groups based on US images. It is closely linked to the presence and severity of MASLD, which is considered a liver manifestation of metabolic syndrome. Recent studies have also supported its use in screening for metabolic syndrome in both healthy and patient populations. However, the LAP has limitations, such as relying on waist circumference measurements, which are not standardized and may not be a robust variable compared to other indices like the FLI [18].

Hepatic steatosis index (HSI): HSI is a biomarker panel that includes BMI, diabetes, and the ALT/AST ratio. It showed an AUC-ROC of 0.79 in the derivation group and 0.82 in the validation group, with cutoffs of 30 and 36 providing over 90% sensitivity and specificity. However, its accuracy decreases in obese children (AUC-ROC of 0.67, sensitivity of 67%, specificity of 62%), and, like FLI, it struggles to differentiate moderate-to-severe steatosis from mild steatosis [19].

SteatoTest: The SteatoTest is a biomarker panel consisting of 10 biochemical tests, age, gender, and BMI. SteatoTest exhibited an AUC-ROC of 0.8 for identifying a greater than 5% liver fat content in patients with chronic liver diseases. Further studies are needed to validate the SteatoTest for differentiating individuals with MASLD from healthy people [19].

Novel MASLD Biomarkers

Identifying noninvasive biomarkers for conditions like simple steatosis, MASH, and fibrosis is crucial, as liver biopsy poses significant risks. Thrombospondin 2, a glycoprotein, shows promise as a noninvasive marker for diagnosing MASH and advanced fibrosis in MASLD, with serum levels correlating with ballooning and fibrosis severity [18]. Metabolomics has identified arachidonic acid oxidation products as potential biomarkers for MASH. Additionally, non-coding RNAs, particularly miR-122, are emerging as stable, noninvasive biomarkers for MASLD detection, aiding in the differentiation of NAFL from MASH and fibrosis stages [18].

Cytokeratin (CK)-18: CK-18 fragments, which result from hepatocyte apoptosis mediated by caspase 3, can be detected in serum through immunoassays. The M30 ELISA measures caspase-cleaved CK-18 fragments, indicating apoptosis, a key feature of steatohepatitis, while the M65 ELISA detects total cell death. Multiple studies have supported these findings, though they have typically involved small populations [19]. CK-18 has several limitations, including the lack of a commercially available clinical test, limited sensitivity at the individual level, and variability in suggested cutoffs and diagnostic accuracy across studies. These challenges make it difficult to determine the appropriate threshold, limiting its clinical utility [22].

Imaging Modalities

Although liver biopsy remains the gold standard for diagnosing MASLD, its invasive nature limits its widespread use. Imaging techniques offer noninvasive alternatives to assess hepatic fat content and fibrosis, making them valuable for screening, diagnosis, and monitoring disease progression [23].

Ultrasonography (US): US is widely available and noninvasive, making it suitable for large-scale MASLD screening. It is the first-line imaging test for suspected MASLD, showing a hyperechogenic liver. A recent meta-analysis found that US had a sensitivity of 85% and specificity of 94% for moderate-to-severe steatosis compared to histology. However, conventional US can only detect liver fat levels of 20% or higher, missing cases with fat content as low as 5% [19]. The accuracy of US in detecting liver fat is reduced in obese and severely fibrotic patients. To address this, US-based scoring systems were developed, showing improved sensitivity for detecting steatosis with less than 20% liver fat. Both semi-quantitative and quantitative systems outperformed conventional US, especially in overweight and obese individuals. The quantitative approach, which uses a computer-based hepatic/renal ratio and hepatic attenuation rate, offers the best diagnostic performance with a sensitivity of 95% and specificity of 100% [18,23]. It is widely available and cost-effective, making it suitable for large-scale MASLD screening. It is qualitative and relies on the increased echogenicity of the liver (termed "bright liver"). It is also effective for detecting moderate-to-severe hepatic steatosis (≥30% fat content) but less reliable for mild steatosis. However, it is operator-dependent with significant intra- and inter-observer variability. Advanced techniques like computer-assisted quantitative US (hepatorenal index) have improved accuracy [23].

Computer tomography (CT): Like US, CT is widely available, easy to perform, and highly accurate for diagnosing steatosis [18]. It uses Hounsfield unit (HU) values to assess liver attenuation, where fat lowers liver density. Various criteria for defining steatosis have been proposed, with hepatic HU less than 40 or a liver HU-spleen HU difference of less than -10 showing sensitivity ranging from 46% to 72% and specificity from 88% to 95%. Like US, CT has limited sensitivity for detecting mild steatosis (<30% liver fat) in clinical settings [23,24]. Additionally, radiation exposure is a drawback of CT use, making it unsuitable for long-term monitoring or use in children. Dual-energy CT has been explored but has not demonstrated significant clinical advantages over standard CT [23].

Controlled attenuation parameter (CAP): The measurement of CAP by transient elastography has become a preferred tool for assessing liver steatosis due to its high diagnostic accuracy and point-of-care capability. It quantifies US attenuation, which is influenced by fat, using the Fibroscan M (3.5 MHz) or XL (2.5 MHz) probes over a small volume of liver tissue [24]. A recent meta-analysis showed that CAP's diagnostic accuracy for detecting hepatic steatosis in patients with various chronic liver diseases, including MASLD, has a sensitivity of 69%, a specificity of 82%, and an AUC-ROC of 0.823 [24]. CAP levels can increase after eating, and their diagnostic performance is strongly dependent on the operator's skills. Additionally, there is no consensus on the appropriate cutoffs. Despite these limitations, the Asia-Pacific guidelines recommend CAP as a standard tool for diagnosing and screening MASLD due to its low cost and convenience [18].

MRI-based techniques: MRI is a noninvasive, high-resolution imaging method that quantifies liver fat without using ionizing radiation. It has been proposed as an alternative to liver biopsy for diagnosing MASLD, including steatohepatitis and fibrosis. MRI-based methods for liver fat quantification include MRS and MRI-proton density fat fraction (PDFF). MRI-PDFF is an advanced tool that measures liver fat content across the entire liver in an objective, quantitative, and reproducible manner. It works by quantifying the protons in the liver and can assess multiple regions. MRI-PDFF has been validated against liver histology for accuracy [18]. MRI-PDFF is currently the most accurate test for quantifying hepatic steatosis and is considered a promising alternative to liver biopsy. It outperforms CAP in detecting all grades of steatosis in MASLD patients (AUC-ROC of 0.99) [18]. However, its use is limited by factors such as variations in field strength, vendor options, phantoms, high cost, the need for radiological expertise, and the lack of standardized cut-off values for steatosis staging [25]. MRS is considered the gold standard for measuring hepatic TG content, as it can detect even trace amounts of liver fat. It quantifies proton signal intensity from water and fat within a specific area to calculate the fat fraction [24]. A recent meta-analysis showed that MRS has high diagnostic accuracy for detecting mild steatosis (≥5% histological grade) with 89% sensitivity and 84% specificity. However, MRS has limitations, including high cost, time consumption, the need for specialized expertise and equipment, and small measurement volumes, which may lead to sampling errors [23,24].

MRI, particularly chemical-shift imaging, provides accurate fat quantification across the entire liver. T1-independent, T2-corrected multi-echo MRI improves accuracy by correcting for iron overload and water-fat signal interference. It is superior for longitudinal follow-up, making it ideal for monitoring disease progression and response to therapy [23].

Histological Assessment

Liver biopsy is considered the gold standard for diagnosing MASH and MASLD. While noninvasive tests for diagnosing these conditions have improved, their accuracy remains insufficient, particularly for detecting early and intermediate stages of MASH [26]. Liver biopsy is the only reliable method to assess the degree and pattern of steatosis, necroinflammation, and fibrosis. It remains the benchmark for validating other diagnostic tests and clinical methods. Therefore, histopathological analysis of liver biopsy specimens continues to be the definitive way to diagnose MASLD/MASH [26].

Liver biopsy has some limitations, including sampling error, variability between different observers (both inter- and intra-observers), and associated risks and complications. These issues can lead to misdiagnosis and inaccuracies in staging. Studies have also highlighted the uneven distribution of MASH lesions, which can result in variability when evaluating paired biopsies [9].

Advances in management

The management of MASLD/MASH requires a multi-faceted approach. While lifestyle modifications remain fundamental, emerging pharmacological options offer hope for more effective treatment strategies. The diversity of therapeutic targets under investigation reflects the complex pathophysiology of these conditions. As research continues, combination therapies targeting multiple disease pathways may prove most effective in managing this growing health challenge [3].

Lifestyle Interventions

Weight loss is the most effective intervention for MASLD, with studies indicating a dose-dependent relationship between weight reduction and disease remission [25]. Studies have demonstrated that a ≥5% loss of body weight is associated with significant reductions in hepatic steatosis (HS), a ≥7% weight loss is associated with a reduction in hepatic inflammation, and a ≥10% loss is associated with a reduction in fibrosis [27].

Diet plays a crucial role in MASLD management, as excessive fructose consumption contributes to obesity, IR, and disease progression. To minimize these risks, sugar intake should be restricted to less than 10% of total daily calories [28]. On the other hand, an omega-3-rich diet, such as one that incorporates fish and fish oil, can promote fatty acid oxidation, decrease lipid synthesis, and improve overall lipid profiles [29].

Physical activity is another key component of MASLD treatment. Guidelines recommend engaging in aerobic and resistance training for 150-200 minutes per week in three to five sessions [28]. Research suggests that combining diet and exercise is more effective in lowering ALT levels than insulin sensitizers while also enhancing cardiovascular health [30]. However, weight loss should be gradual, as rapid reductions can worsen steatohepatitis and increase the risk of liver failure and gallstones [30]. In addition to lifestyle changes, pharmacological options like orlistat, a lipase inhibitor that blocks fat absorption, and sibutramine, an appetite suppressant, have shown benefits in reducing serum transaminase levels and hepatic steatosis [30].

Despite the proven benefits of lifestyle interventions, long-term adherence is the main challenge. Factors such as socioeconomic status constraints, limited access to healthy food or safe exercise spaces, and low health literacy can undermine sustained behavioral change. Tailoring interventions to personalized individual patient needs by suggesting culturally appropriate diets, supervised exercise programs, behavioral counseling, and digital health tools may enhance compliance and long-term success. A multidisciplinary approach that involves dietitians, exercise physiologists, and psychologists can further support adherence and personalize care plans [30].

Pharmacologic Therapies

Currently, there is no specific drug for the treatment of MASLD, and any medication prescribed for MASLD is considered off-label treatment (Table 2). This should be discussed with the patient before prescription [3].

**Table 2:** MASLD pharmacotherapy TZDs: thiazolidinedione, PPARα: peroxisome proliferator-activated receptor alpha, PPARγ: peroxisome proliferator-activated receptor gamma, SPPARMα: selective peroxisome proliferator-activated receptor alpha modulators, MASLD: metabolic dysfunction-associated steatotic liver disease, T2DM: type 2 diabetes mellitus, TG: triglycerides, HDL-C: high-density lipoprotein cholesterol, ALT: alanine aminotransferase, MASH: metabolic dysfunction-associated steatohepatitis, ASBT: apical sodium-dependent bile acid transporter inhibitor, FFAs: free fatty acids

Drug class	Mechanism
TZDs	These drugs represent the strongest evidence-based treatment currently available. They work by promoting the differentiation of insulin-resistant adipocytes into insulin-sensitive ones, diverting FFAs from the liver to adipose tissue, increasing adiponectin production, which enhances fatty acid oxidation [3]. Pioglitazone, a thiazolidinedione derivative, is a potent activator of the nuclear receptor PPARγ, which plays a crucial role in adipocyte differentiation and the regulation of lipid and glucose metabolism [28]. Although primarily prescribed for diabetes, it has also shown effectiveness in managing MASLD by reducing hepatic steatosis, necroinflammation, and fibrosis [28]. However, it is associated with side effects, including significant weight gain, lower extremity edema, atypical chest pain, and joint pain [30].
GLP-1 agonists	GLP-1 agonists enhance insulin secretion, inhibit glucagon release, and reduce hepatic glucose production [31]. They also suppress appetite and promote weight loss, making them particularly beneficial for obese individuals with MASLD [31]. Clinical studies have shown that liraglutide, a GLP-1 agonist used for T2DM, effectively improves glycemic control, lowers liver enzyme levels, and reduces hepatic steatosis, contributing to MASH resolution [18]. Semaglutide, another GLP-1 agonist approved for diabetes treatment, shares a similar mechanism with liraglutide but has demonstrated superior metabolic effects [30]. Given the strong link between diabetes, MASLD, and metabolic syndrome, GLP-1 agonists offer a promising therapeutic option due to their efficacy, safety, and tolerability [18]. In a comparative trial, semaglutide led to greater weight loss and more substantial improvements in cardiometabolic parameters than liraglutide [32]. Common side effects of GLP-1 agonists include nausea, vomiting, diarrhea, and constipation [18].
SGLT-2 inhibitors	SGLT-2 inhibitors, a newer class of antidiabetic medications, lower blood glucose levels by reducing renal glucose reabsorption, resulting in significant glycosuria [18]. While SGLT-2 inhibitors are not currently approved specifically for MASLD, emerging evidence supports their potential role in managing this condition, particularly in individuals with T2DM. Some examples, such as dapaglifozin and canagliflozin, have shown hepatic fat content and improvements in liver function markers in patients with T2DM and MASLD. Moreover, Empagliflozin has been able to reduce fat content in patients with and without T2DM [33]. While SGLT-2 inhibitors are primarily used for glycemic control, they also promote weight loss and lower blood pressure, similar to GLP-1 agonists [18]. However, their use is associated with side effects such as polyuria due to increased diuresis and an elevated risk of fungal genitourinary infections. Additionally, they may contribute to acute kidney injury and euglycemic diabetic ketoacidosis [18].
MSDC-0602K	This second-generation insulin sensitizer shows promise in enhancing lipid oxidation. Reducing de novo lipid synthesis and improving hepatic histology without the side effects associated with first-generation drugs [3].
Selective PPARα modulator	SPPARMα regulates lipid and lipoprotein metabolism by transcriptionally modulating genes involved in reducing serum TG and increasing HDL-C. Clinical studies indicate that pemafibrate demonstrates superior efficacy in lowering serum TG levels and raising HDL-C while also offering an improved safety profile [18]. In a study involving 16 patients treated with 200 mg/day of fenofibrate for 48 weeks, significant reductions were observed in TGs, glucose, and gamma-glutamyl transpeptidase levels, as well as in the proportion of patients with elevated transaminases or metabolic syndrome. There was also a trend toward decreased IR. Repeat liver biopsies showed reduced hepatocellular ballooning, although other histologic features did not show significant improvement [34]. There was no proven advantage with clofibrate in patients with MASLD. Newer selective PPARα-specific agonists, known as selective PPARα modulators, are in different phases of development [35]. However, their use is associated with side effects such as polyuria due to increased diuresis and an elevated risk of fungal genitourinary infections. Additionally, they may contribute to acute kidney injury and euglycemic diabetic ketoacidosis [18].
Vitamin E	Vitamin E helps reduce oxidative stress, a key contributor to MASH progression, making it a potential treatment option for MASLD [18]. Clinical trials indicate that while it improves liver function and lowers oxidative stress, it does not significantly enhance histological grade [36]. In a study comparing vitamin E alone to a combination of vitamin E and pioglitazone in patients without T2DM, both groups showed reduced serum ALT levels; however, significant histological improvement was observed only in the combination group [18]. Despite its potential benefits, vitamin E supplementation carries risks, including an increased likelihood of all-cause mortality, bleeding, heart failure, and hemorrhagic stroke [18]. Long-term safety concerns exist, particularly regarding cardiovascular effects and prostate cancer risk in older males [3].
Novel therapeutic targets	
FXR agonists	Obeticholic acid and other FXR agonists represent a promising therapeutic avenue by regulating bile acid synthesis, improving glucose homeostasis, reducing inflammation, and fibrosis [3]. Obeticholic acid, elafibranor, selonsertib, and cenicriviroc are currently undergoing phase III randomized controlled trials to evaluate their potential therapeutic benefits for MASLD [30]. Several phase II and III studies have already assessed the efficacy and safety of these new agents in treating MASLD and MASH, showing promise as potential treatment options [30].
Thyroid hormone receptor-β agonists	These compounds, including Resmetirom and VK2809, show potential in reducing hepatic fat content, improving lipid profiles, and maintaining tissue selectivity to minimize side effects [3].
AZD2693	AZD2693, an antisense oligonucleotide, represents a significant advancement in precision medicine for MASH, offering targeted therapy for patients with the PNPLA3 p.I148M genetic variant [37]. This GalNAc-conjugated antisense oligonucleotide has demonstrated compelling early-phase clinical results, achieving robust target engagement with an 89% reduction in hepatic PNPLA3 mRNA and 63% reduction in protein expression at the 80 mg dose. The therapeutic potential is further underscored by meaningful clinical outcomes, including a placebo-corrected 12.2% reduction in liver fat at 12 weeks with the 50 mg dose, alongside notable anti-inflammatory effects evidenced by reductions in high-sensitivity C-reactive protein (up to 37%) and interleukin-6 [37]. The drug's favorable safety profile, characterized by mild to moderate adverse events and absence of treatment-related serious events or discontinuations, supports its continued development despite dose-dependent transaminase elevations that remained within acceptable thresholds [37]. While the phase I data are limited by small sample sizes and short duration, the consistent pharmacodynamic effects, favorable pharmacokinetics supporting monthly dosing, and biologically rational mechanism targeting a well-validated genetic risk factor position AZD2693 as a promising therapeutic option. The ongoing phase IIb FORTUNA trial will provide crucial histological endpoints to further validate this precision approach to treating genetically defined MASH patients, potentially addressing a significant unmet medical need in hepatology [37].
Volixibat	Volixibat, a selective ASBT inhibitor, demonstrated a disconnect between pharmacodynamic activity and clinical efficacy in MASH treatment [38]. Despite successfully engaging its target, evidenced by increased serum C4 and reduced cholesterol levels, the drug failed to improve liver fat content, ALT levels, or histological outcomes at 48 weeks across all tested doses. While generally well-tolerated, gastrointestinal adverse events, particularly diarrhea affecting 74% of patients, led to significant discontinuations. The study's early termination underscores that ASBT inhibition alone represents an insufficient therapeutic strategy for MASH, though combination approaches may warrant future investigation [38].
Ervogastat	Ervogastat is a diacylglycerol O-acyltransferase 2 inhibitor evaluated in combination with the acetyl-CoA carboxylase inhibitor clesacostat for treating MASH. In the MIRNA phase II trial, this dual therapy targeted hepatic lipid metabolism to reduce steatosis and fibrosis [39]. While early-phase results confirmed target engagement and biochemical improvements, histologic efficacy data remain pending. The combination was generally well tolerated, with manageable adverse events. Although promising in mechanism, its clinical reliability awaits validation through long-term, placebo-controlled trials with histologic endpoints to confirm sustained efficacy and safety in broader MASH populations [39].
Cytokine-based approaches	Understanding the role of various cytokines has opened new therapeutic possibilities. TNF-α inhibition shows promise in reducing inflammation, IL-11 pathway modulation may help reverse liver fibrosis, and TGF-β targeting could reduce fibrosis progression [3].
Gut microbiome modulation	The gut-liver axis has emerged as a crucial therapeutic target. Approaches include probiotics and prebiotics to improve metabolic function; fecal microbiota transplantation shows early promise in improving insulin sensitivity and symbiotic combinations demonstrating effects on inflammation and lipid metabolism [3].

Bariatric Surgery

For patients who do not respond to lifestyle modifications and pharmacotherapy, bariatric surgery is a viable option for sustained weight loss and metabolic improvement [40]. Bariatric surgery is not yet formally approved exclusively for MASLD/MASH, but it is considered an effective treatment in appropriate patients. It has been shown to improve liver histology by reducing steatosis, ballooning, and fibrosis [40]. While the expected weight loss varies depending on the type of surgery, patients typically lose 50-80% of their excess body weight within 12-18 months [10]. Bariatric surgery is generally recommended for patients with a BMI ≥40 kg/m² or ≥35 kg/m² in the presence of obesity-related comorbidity such as T2DM, hypertension, or obstructive sleep apnea. Among surgical options, sleeve gastrectomy is the most commonly performed procedure [18]. Despite its benefits, bariatric surgery has not been widely recognized as a therapeutic option for MASLD, and therefore, further research is required in this field. However, combining pharmacological treatment with bariatric surgery may yield better outcomes [18]. A recent meta-analysis found that pioglitazone, when administered alongside Roux-en-Y gastric bypass surgery, resulted in greater improvements in MASLD activity [18].

For severely obese patients, bariatric surgery offers benefits beyond weight loss. It improves metabolic parameters, reduces liver inflammation, and may reverse fibrosis in some cases. However, careful patient selection is crucial due to potential complications [3]. While bariatric surgery is generally effective, a small number of patients have experienced worsening liver disease, including the development of MASH, hepatic decompensation, and, in rare cases, the need for liver transplantation [40].

Prognosis

The MASLD spectrum includes simple steatosis, which is relatively benign, and MASH, which carries a higher risk of fibrosis, cirrhosis, liver failure, and HCC. The prognosis of MASLD is largely dependent on the histological stage of fibrosis. Simple steatosis exhibits minimal risk of progression and is considered benign, whereas progression to MASH poses a significant threat due to its propensity for fibrotic transformation [20]. Advanced fibrosis (stages F3-F4) is the strongest predictor of liver-related and overall mortality. Given the silent nature of MASLD, early detection of high-risk individuals is crucial to prevent disease progression [20]. Key aspects of prognosis in MASLD include a markedly higher risk of liver-related mortality in patients with advanced fibrosis (F3-F4) [20]. Cardiovascular disease remains the leading cause of death in these patients, surpassing liver-related mortality [20]. Additionally, individuals with MASLD-related cirrhosis face an increased risk of developing HCC, which can also occur in the absence of cirrhosis in some cases [20]. Overall survival is further compromised by the presence of metabolic syndrome and diabetes, both of which significantly elevate mortality risk [23].

Prognostic Factors

Several factors influence the progression of MASLD, with the fibrosis stage being the most significant. Fibrosis is the strongest predictor of adverse outcomes in MASLD, significantly impacting both liver-related and cardiovascular mortality. Patients with mild fibrosis (F0-F1) have a lower risk but still require monitoring due to the potential for progression to severe disease [20]. Meanwhile, F2 (moderate fibrosis) has an intermediate risk of progression, requiring lifestyle interventions and regular monitoring. Fibrosis progression, particularly to F3-F4, is associated with increased morbidity and mortality. F3 (advanced fibrosis) is associated with an increased risk of cirrhosis, hepatic decompensation, and HCC. F4 (cirrhosis) has an increased risk of HCC, liver failure, and transplantation [20].

Fibrosis progression can occur in up to 30-40% of MASLD patients over a 5-10 year period. Studies suggest that one in five MASLD patients with mild fibrosis can progress to cirrhosis within a decade [20]. The distribution of fibrosis stages follows a characteristic pattern, with 50% of cases presenting at F1 (portal/centrilobular), while more advanced stages (F2-F4) collectively represent approximately 27% of cases. The progression rate, estimated at 6% advancing to severe fibrosis within a decade, raises concerns about the potential need for liver transplantation in young adulthood [21].

Metabolic Risk Factors

Obesity and IR are strongly associated with fibrosis progression. T2DM increases the risk of fibrosis progression, cirrhosis, and mortality. Dyslipidemia and hypertension are linked to faster disease advancement [20].

Age and Gender

Older age and male gender are associated with a higher risk of fibrosis progression [20]. Although MASLD is more often present in older adults, it has emerged as a significant health concern in pediatric populations, presenting unique challenges in diagnosis and management. Recent studies have revealed an intriguing inverse relationship between age and disease severity, with prepubertal patients showing more advanced fibrosis patterns. The median diagnostic age of 13.0 years (range: 11-16) underscores the importance of early recognition and intervention [21]. Pediatric MASLD represents a complex clinical challenge requiring vigilant monitoring and comprehensive assessment.

Male patients demonstrate notably higher susceptibility to advanced disease states, with 82% showing some degree of fibrosis compared to 69% in females. This disparity becomes even more apparent in moderate fibrosis cases, where males show a 30% prevalence rate versus 21% in females. Importantly, age-dependent progression demonstrates statistical significance in males (p=0.001) but not in females, suggesting distinct pathophysiological mechanisms between genders [21].

*Noninvasive M* *arkers*

Noninvasive markers provide prognostic insights. Some of these markers are presented in Table 3.

**Table 3:** Noninvasive markers of MASLD prognosis NFS: non-alcoholic fatty liver disease fibrosis score, FIB-4: fibrosis-4, APRI: alanine aminotransferase-to-platelet ratio index, SAF: steatosis activity and fibrosis, ELF: enhanced liver fibrosis, FAST: FibroScan-aspartate aminotransferase, MEFIB: magnetic resonance elastography and fibrosis-4, BMI: body mass index, AST: aspartate aminotransferase, ALT: alanine aminotransferase, AUC-ROC: area under the receiver operating characteristic curve, MASH: metabolic dysfunction-associated steatohepatitis, HA: hyaluronic acid, MASLD: metabolic dysfunction-associated steatotic liver disease, LSM: liver stiffness measurement, CAP: controlled attenuation parameter, ADAPT: age, diabetes, platelet count, and PRO-C3, NPV: negative predictive value, GGT: gamma-glutamyltransferase

Maker	Indicators	Interpretation
NFS	Uses age, BMI, diabetes, AST/ALT ratio, platelet count, and albumin	NFS < -1.455 suggests low risk of fibrosis; NFS > 0.676 suggests high risk of advanced fibrosis [20]
FIB-4 index	Based on age, AST, ALT, and platelet count	FIB-4 ≥ 2.67 suggests advanced fibrosis; FIB-4 ≤ 1.30 suggests low risk [20]
APRI	APRI demonstrates the highest reliability for advanced fibrosis detection (AUC-ROC 0.759), offering a strong negative predictive value of 94%. However, its limited sensitivity (55%) constrains its utility as a standalone prognostic tool [21]	Less accurate than NFS or FIB-4 but can still help in fibrosis risk stratification [20]
SAF score	SAF score distinguishes fat accumulation from inflammation, recognizing their distinct prognostic significance. MASH is diagnosed when steatosis is present, and both ballooning and inflammation score at least 1. Notably, patients with advanced fibrosis but minimal fat or inflammation may still be categorized as having significant diseases [20]	The SAF score is a validated system for classifying MASLD based on steatosis (0-3), activity (ballooning 0-2, lobular inflammation (0-2), and fibrosis (0-4). Disease severity is classified as mild (A < 2, F < 2) or significant (A ≥ 2, F ≥ 2), emphasizing the role of fibrosis in long-term outcomes [20]. While the SAF score provides a more comprehensive assessment, its correlation with long-term liver-related mortality remains under investigation [20]
BARD score	Considers BMI, AST/ALT ratio, and diabetes	A score of 2-4 increases the likelihood of advanced fibrosis [20]
ELF test	Measures TIMP-1, HA, and P3NP	Accurate for detecting advanced fibrosis [20]
FAST score	FAST score, a noninvasive diagnostic tool designed to identify patients with MASH who have significant disease activity (MASLD activity score ≥4) and fibrosis stage ≥2 [41]. Combining LSM, CAP, and AST levels, the FAST score enables simultaneous assessment of steatosis, inflammation, and fibrosis [41]	In an Indian cohort study, the FAST score demonstrated strong diagnostic performance (AUC-ROC 0.81) [39]. Locally optimized cut-offs improved accuracy over standard ones, yielding a negative predictive value of 0.95 and a positive predictive value of 0.70. It is particularly effective for ruling out advanced MASH, minimizing unnecessary biopsies [41]
MEFIB index	-	Improves detection of at-risk MASH cases [32]
PRO-C3	PRO-C3 is a serological biomarker reflecting the formation of type III collagen, a key component of hepatic fibrogenesis. It is particularly useful in diagnosing and assessing fibrosis severity in chronic liver diseases, including MASH [42]. PRO-C3 levels correlate strongly with fibrosis stage. Elevated levels are significantly associated with advanced fibrosis (F3–F4) in both MASH and alcohol-related liver disease [42]. PRO-C3 can complement liver function tests to enhance early detection of fibrosis, offering a noninvasive alternative to liver biopsy [42]	High PRO-C3 indicates increased fibrotic activity and worse clinical outcomes, making it a promising marker for disease progression monitoring [42]. PRO-C3 demonstrated good diagnostic accuracy with an AUC-ROC of 0.85 for detecting advanced fibrosis. When integrated into the ADAPT algorithm, the diagnostic performance improved significantly (AUC-ROC: 0.88), outperforming traditional non-patented fibrosis tests like FIB-4 and forns index [42]. PRO-C3 showed an NPV of 95-97%, making it highly reliable in ruling out advanced fibrosis, especially in primary care or low-risk settings. One limitation is that false positives can occur, particularly in patients with elevated GGT levels, which should be considered during interpretation [42]. A PRO-C3 level below 15.6 ng/mL or an ADAPT score below 6.3 reliably excludes advanced fibrosis, suggesting a potential role in routine liver disease screening [42]

Imaging for Fibrosis and NASH Prognosis

Since fibrosis is a key determinant of MASLD prognosis, imaging techniques that assess liver stiffness have become increasingly important.

US elastography: US elastography measures liver stiffness as a surrogate for fibrosis. Techniques include transient elastography (FibroScan), acoustic radiation force impulse, and supersonic shearwave elastography [23]. Highly accurate for advanced fibrosis detection but less reliable for mild fibrosis or distinguishing MASH from simple steatosis [23]. Transient elastography (FibroScan) faces significant limitations in MASLD assessment, with failure rates of 14-17% using standard M-probes, primarily due to invalid measurements in patients with elevated BMI and central obesity. However, utilization of XL-probes substantially improves diagnostic success, reducing failure rates to below 2% in this challenging patient population [20].

Vibration-controlled transient elastography (FibroScan): Liver stiffness measurement (LSM) >12 kPa indicates advanced fibrosis. Widely used for serial fibrosis monitoring [43].

Magnetic resonance elastography: MRE is more precise than US elastography in staging liver fibrosis. It can detect early fibrosis and differentiate MASH from simple steatosis based on increased liver stiffness in inflamed livers. It is the most accurate imaging technique for fibrosis detection: LSM >3.6 kPa correlates with significant fibrosis [43]. However, MRE is more expensive and less widely available compared to US elastography [23].

Fat Quantification Techniques

MRI-PDFF measures hepatic fat content and tracks steatosis resolution [43]. CAP (FibroScan) assesses liver fat burden [43].

US remains the first-line imaging tool for MASLD screening. MRI and MRS are the most accurate methods for fat quantification, especially for clinical trials and monitoring treatment response. Elastography techniques (US and MRE) are crucial for fibrosis staging and prognosis assessment [23]. Noninvasive imaging techniques could potentially replace liver biopsy as reference standards in MASLD research. Emphasizing MRI and elastography as the most promising modalities for accurately assessing liver fat and fibrosis. While US and CT are useful for initial detection, MRI-based methods offer the most precise, reproducible, and clinically valuable assessments for disease monitoring and prognosis [23].

Genetic Predisposition

PNPLA3 (I148M variant) is associated with higher fibrosis severity and faster progression; TM6SF2 and MBOAT7 polymorphisms influence hepatic fat accumulation and fibrosis risk, while HSD17B13 mutation may offer protective effects against fibrosis progression [20].

## Conclusions

MASLD is a rapidly evolving public health concern with significant metabolic and liver-related morbidity and mortality. This review highlights its epidemiology, pathophysiology, diagnostic advancements, and management strategies, emphasizing the importance of early detection and targeted interventions. While lifestyle modifications remain the cornerstone of treatment, emerging pharmacological and surgical options provide additional therapeutic avenues. Advances in imaging and biomarker research offer promising alternatives to invasive liver biopsy, facilitating early diagnosis and disease monitoring. This review is limited by the rapid evolution of MASLD research, which may result in some recent advancements not being fully captured. Nonetheless, key aspects of MASLD are integrated in this review, underscoring the need for personalized, multi-faceted management strategies.

Future research should prioritize improving noninvasive diagnostic methods, confirming the effectiveness of emerging therapies, and understanding individual differences in disease progression and treatment response to enhance patient outcomes. Additionally, more longitudinal studies are needed in pediatric populations, where data remain limited despite the growing impact of MASLD among children.
